# Emergence of lung cancer with a low PD-L1 expression level after the administration of immune check point inhibitor for lung adenocarcinoma with a high PD-L1 expression level: A case report

**DOI:** 10.1016/j.amsu.2020.05.021

**Published:** 2020-05-23

**Authors:** Shun Iwai, Aika Funasaki, Atsushi Sekimura, Nozomu Motono, Katsuo Usuda, Sohsuke Yamada, Yoshimichi Ueda, Kyouta Akasaki, Kouta Tanimura, Kazumasa Kase, Hidetaka Uramoto

**Affiliations:** a Department of Thoracic Surgery, Kanazawa Medical University, 1-1 Daigaku, Uchinada, Ishikawa, 920-0293, Japan; b Department of Clinical Pathology & Laboratory Medicine, Kanazawa Medical University, 1-1 Daigaku, Uchinada, Ishikawa, 920-0293, Japan; c Department of Pathology, Kanazawa Medical University, 1-1 Daigaku, Uchinada, Ishikawa, 920-0293, Japan; d Department of Internal Medicine, Keiju Medical Center, 94 Tomioka, Nanao, Ishikawa, 926-8605, Japan

**Keywords:** Immune check point inhibitor, PD-L1, Lung cancer, resistance, VATS

## Abstract

**Background:**

Checkpoint therapy against PD-1 has proven effective and positive results have been observed in several types of cancer, including lung cancer, renal cancer, lymphoma and melanoma. However, the effects of long-term ICI treatment remain insufficient and the development of resistance is an issue that remains to be solved.

**Case presentation:**

A 70-year-old man was diagnosed with lung adenocarcinoma (stage IVB, T4N3M1c) with a high programmed death ligand-1 (PDL1) expression level (tumor proportion score [TPS]: 80% score at the time of the diagnosis, before treatment). At 16.5 months after the start of pembrolizumab, following the administration of 22 cycles of pembrolizumab, a new lesion appeared. Biopsy by video-assisted thoracic surgery (VATS) was performed for this lesion and a pathological diagnosis of lung adenocarcinoma with a low PD-L1 expression level. After the operation, pembrolizumab treatment was continued. The patient currently remains alive without disease progression at 20 months after the initial therapy.

**Conclusions:**

Our case highlights the importance of biopsy by VATS during immune checkpoint inhibitor (ICI) treatment when deciding the treatment strategy for newly confirmed tumors.

## Highlights

• The importance of biopsy by VATS during ICI treatment when deciding the treatment strategy for newly confirmed tumors.

## 1 Introduction

Lung cancer is still commonly diagnosed at a late stage. Immune checkpoint inhibitors, which have recently become available, assert antitumor effects using the immune system. Inhibition of PD-1 has shown positive results in several types of cancer, including lung cancer, renal cancer, lymphoma, and melanoma [1]. Pembrolizumab is a monoclonal antibody designed to identify and block the PD-1 receptor [2]. In early reports, high expression of PD-L1 (≥50%) was reported to be associated with a higher partial response (PR), longer progression free survival (PFS), and longer overall survival (OS) when administered as second-line treatment for patients who had previously received chemotherapy [3]. Recently, the phase III KEYNOTE-042 trial demonstrated that pembrolizumab significantly improved PFS and OS compared to platinum-based chemotherapy as first-line therapy for advanced or metastatic NSCLC with a PD-L1 TPS of ≥1% [4]. However, the effects of long-term ICI treatment remain insufficient, and the development of resistance needs to be addressed.

We present a case in which lung cancer with low PDL-1 expression emerged after the administration of immune checkpoint inhibitors for lung adenocarcinoma with high PD-L1 expression. The present case has been reported in line with the SCARE criteria [5].

## 2 Presentation of case

**Fig. 1:**
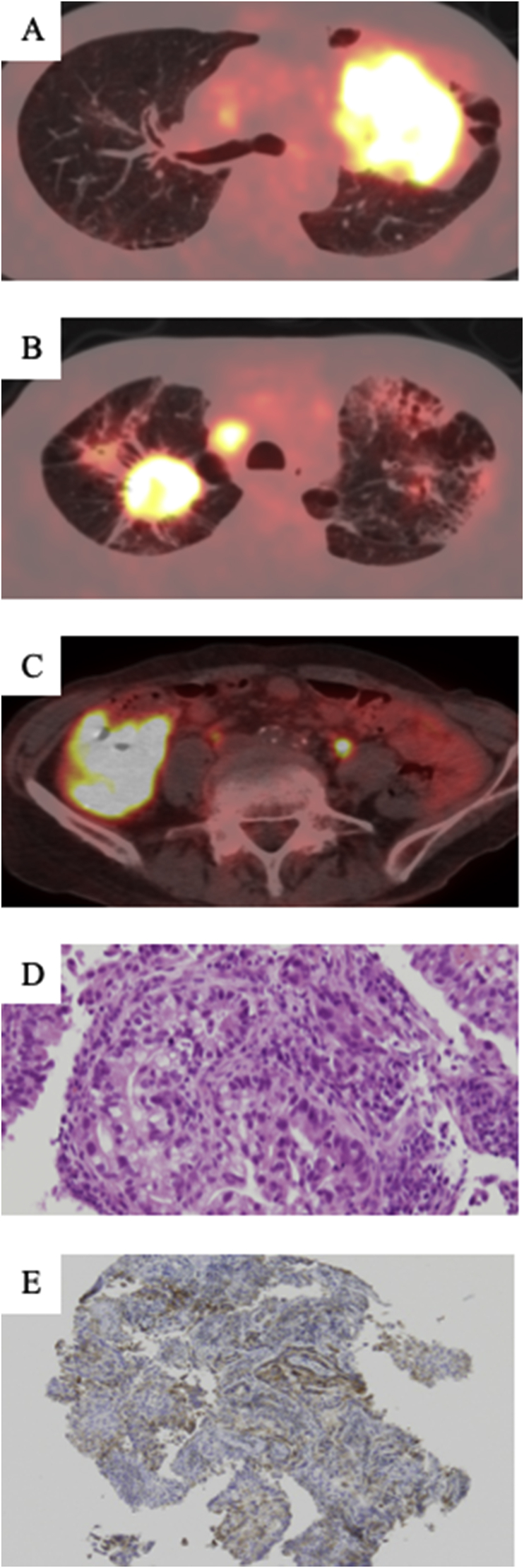
A: PET-CT on admission showing a 95-mm primary lung tumor in the left upper lobe with pulmonary metastasis in the right upper lobe. B: A-45 mm lesion in the right upper lobe with contralateral mediastinal lymph node enlargement. C: Primary colon cancer. D: The histopathological findings of the lung specimens. Hematoxylin and eosin staining of the lung tissue obtained by transbronchial lung biopsy showing moderately differentiated adenocarcinoma. D: Immunohistochemical staining of PD-L1 the high PD-L1 expression of tumor cells (TPS: 80%).

**Fig. 2:**
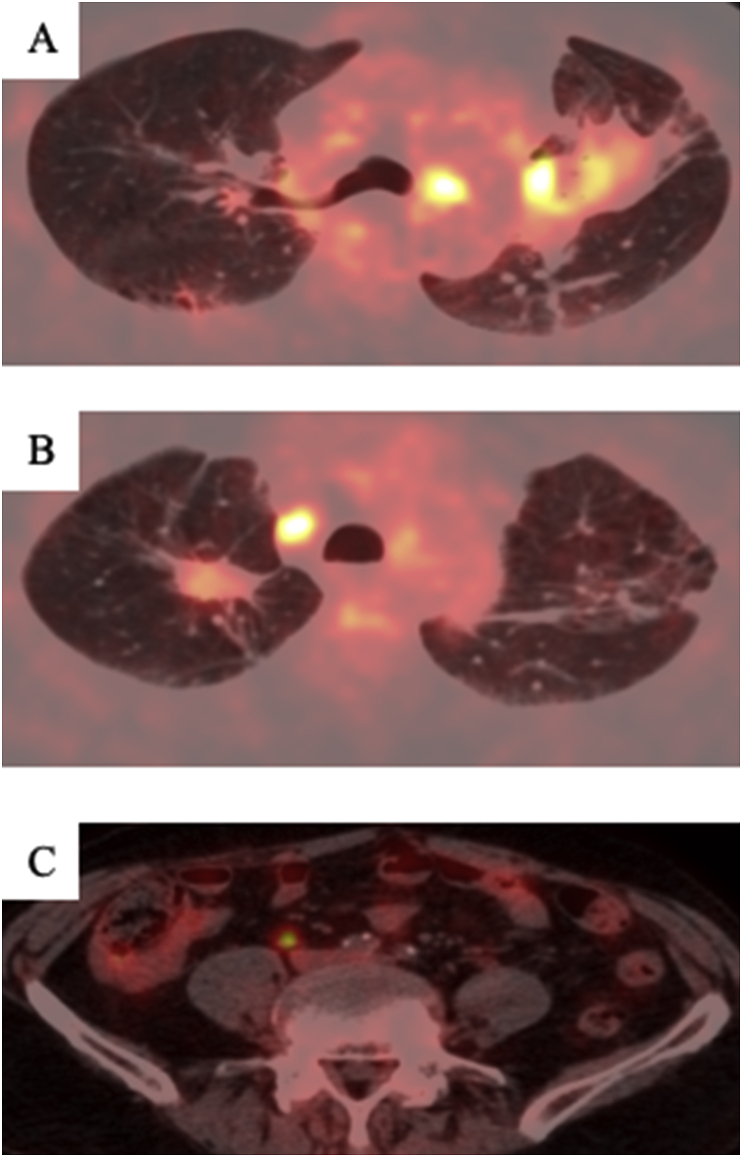
A and B: The primary lung tumor, pulmonary metastasis, and contralateral mediastinal lymph node enlargement was observed to have shrunk remarkably after 22 cycles of pembrolizumab. C: The primary colon tumor also showed a partial response.

**Fig. 3:**
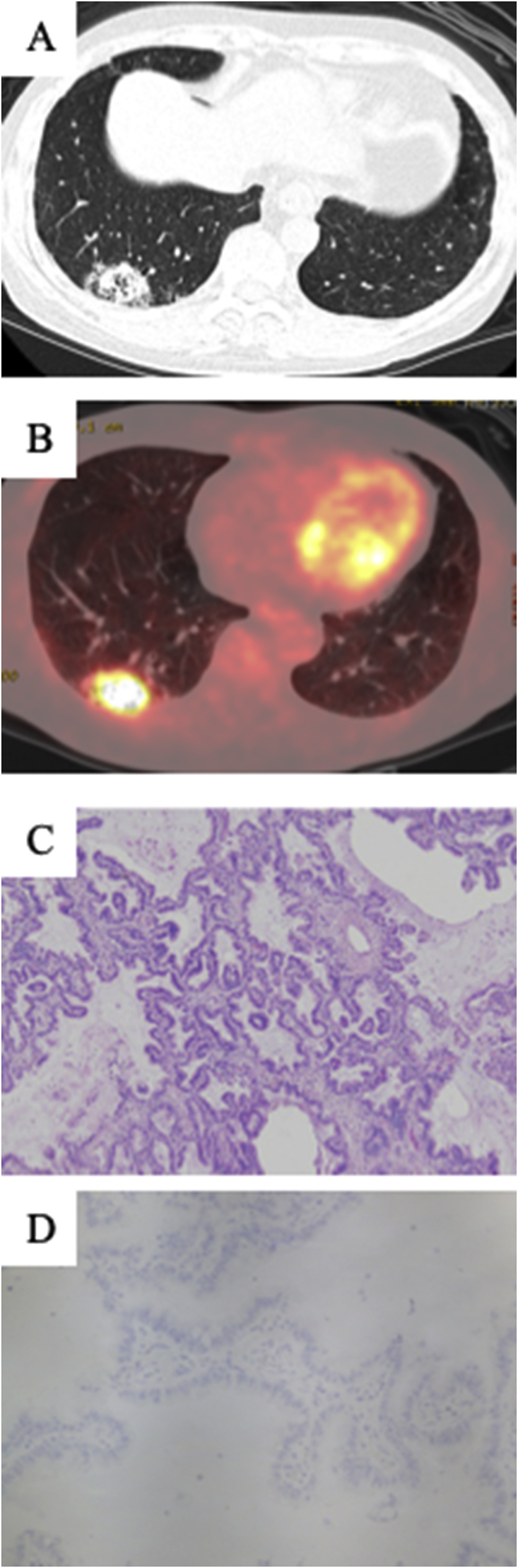
A: A new lesion in the right lower lobe (RLL) was detected on CT. B: A PET-CT revealed an increased fluorodeoxyglucose uptake (FDG) in the RLL. C: Hematoxylin and eosin staining of the lung tissue, obtained by VATS, revealed a histopathological diagnosis of invasive mucinous adenocarcinoma. D: Immunohistochemical staining of PD- L1. The tumor cells showed low PD-L1 expression levels (TPS: 1%).

A 70-year-old man with a 100-pack-year smoking history presented with a cough. Positron emission tomography-computed tomography (PET-CT) revealed a 95 mm tumor in the left upper lobe of the lung (Fig. 1A), bilateral pulmonary metastases with contralateral mediastinal lymph node enlargement (Fig. 1B), contralateral adrenal gland metastasis, and primary colon cancer (mucinous carcinoma) (Fig. 1C). Brain magnetic resonance imaging revealed no metastatic lesions. His serum CEA and CYFRA levels were elevated (25.9 ng/mL [normal <5.0 ng/mL] and 14.7 U/mL [normal <3.5 U/mL], respectively). Bronchoscopic biopsy was performed targeting the mass in the left upper lobe, and the tumor was histologically diagnosed as moderately differentiated adenocarcinoma (T4N3M1c, stage IVB) (Fig. 1D). The tumor harbored neither *EGFR* mutation nor *ALK* rearrangement. The tumor cells strongly expressed PD-L1, and the TPS was 80% (Fig. 1E). Therefore, pembrolizumab was introduced as first-line therapy. After four cycles of pembrolizumab without any adverse effects, the left lung tumor, pulmonary metastases, hilar and mediastinal lymphadenopathy, and primary colon cancer notably decreased. PET-CT of the primary lung and colon cancer indicated PR according to the RECIST criteria (Fig. 2). However, a new lesion in the right lower lobe(RLL) was detected on PET-CT after 22 cycles of pembrolizumab (Fig. 3A and B). It was unclear whether this lesion represented a benign disease (e.g., interstitial pneumonia or a fungal infection), a malignant tumor (e.g., double primary lung cancer or pulmonary metastasis [PM]), or another disease. We considered a definitive etiologic diagnosis to be necessary. Although transbronchial lung biopsy and CT-guided needle biopsy were performed, a definitive diagnosis was not made. No fungi or bacteria were detected, and acid-fast bacteria staining of bronchial washing fluids was negative. Lung biopsy was performed by VATS to make a histological diagnosis.

A pathological examination confirmed invasive mucinous adenocarcinoma without EGFR mutation (Fig. 3C), and the PD-L1 TPS was only 1% (Fig. 3D). The new lesion in the RLL was diagnosed as an emergence of new lung cancer based on the comparison of the histological diagnosis and immunohistochemical analysis of the tumors (Table 1). After surgery, pembrolizumab treatment was continued because of the efficacy of local treatment. The patient currently remains alive without disease progression at more than 20 months after the initial therapy.

**Table 1:** Comparison of histologically diagnosis and immunohistochemical analysis for tumors.

Lesion	histological diagnosis	PD-L1 status (TPS)	TTF-1	CK7	CK20	CDX2	ALK	P40	NapsinA
Primary lung cancer	Moderately differentiated adenocarcinoma	80%	+	+	–	–	–	–	+
Primary colon cancer	Mucinous carcinoma	n.d	–	+	–	–	–	n.d	n.d
Second lesion	Invasive mucinous adenocarcinoma	1%	+	+	+	–	–	n.d	+

TPS: tumor proportion score, n.d: Not done.

## 3 Discussion

The present case raises two important points. The first is in relation to whether the new lesion represented a malignant or benign tumor. If it was a malignant tumor, it would be necessary to suspect PM, from the left lung cancer or multiple lung cancer. If this case was diagnosed as PM based on the pathological diagnosis, then it would be necessary to change treatment. However, interestingly, the new lesion in the RLL was diagnosed as an emergence of multiple lung cancer based on the pathological diagnosis. When local therapy, in this case surgery, was properly performed it was possible to continue pembrolizumab.

The second point is in relation to resistance against ICI therapy. There is a possibility that the first tumor had heterogeneous PD-L1 expression, and the tumor with low PD-L1 expression arose during the administration of pembrolizumab as cells with high PD-L1 expression were eradicated. This is observed not only in the main tumor but also between different lesions [6]. Lung adenocarcinoma that is immunohistochemically negative for PD-L1 has been reported after the administration of crizotinib for a tumor with high PD-L1 expression (TPS 90%) [6]. In our case, pembrolizumab was administered as first-line therapy for advanced NSCLC with high PD-L1 expression (TPS 80%). All of the lesions shrank (including the colon cancer lesion) and PR was obtained. The PD-L1 status and microsatellite instability, which are therapeutic markers for colorectal cancer , remain unexplored [7] in the colon cancer specimens of cases of double primary lung and colon adenocarcinomas, as in this case. Such cases suggest that double primary cancer consisting of colon and lung tumors with multiple lung cancer may occur with transforming KRAS mutations [8]. In this case, pathological examination confirmed that the new primary lung tumor was an invasive mucinous adenocarcinoma without EGFR mutation. We previously reported that EGFR mutations and KRAS mutations are mutually exclusive, suggesting the presence of several distinct mechanisms in the development of multiple cancers [9]. However, a high tumor mutation burden (TMB), which is considered to be a new predictive biomarker for identifying patients who are likely to benefit from ICI therapy, might be present in patients with double cancer. Our patient was previously a heavy smoker, which is associated with a high TMB [10].

## 4 Conclusion

Immune checkpoint inhibitor therapy is considered to have potential as a future treatment approach; however, it is possible that many cases similar to ours will emerge. Moreover, it is necessary to differentiate between recurrence and second primary lung cancer. In summary, a careful diagnosis should be considered after performing the biopsy. We believe that biopsy of newly confirmed tumors will become an essential diagnostic examination in patients receiving immune checkpoint inhibitor treatment.

## Consent to publication

Consent for publication was obtained from the patient.

## Funding

No funding was obtained for this study.

## Guarantor

Shun Iwai, Kanazawa Medical University, 1-1 Daigaku, Uchinada, Ishikawa, 920-0293, Japan.

## Abbreviations

PD-1: lung cancer receptor
PD-L1: programmed death ligand-1
PET-CT: positron emission tomography-computed tomography
EGFR: epidermal growth factor receptor
PR: partial response
PFS: progression-free survival
OS: overall survival
PM: pulmonary metastasis
ICI: immune checkpoint inhibitor
TMB: tumor mutation burden
NSCLC: non-small-cell lung carcinoma
RLL: right lower lobe
